# Candidate genes and SNPs associated with stomatal conductance under drought stress in *Vitis*

**DOI:** 10.1186/s12870-020-02739-z

**Published:** 2021-01-06

**Authors:** Massimiliano Trenti, Silvia Lorenzi, Pier Luigi Bianchedi, Daniele Grossi, Osvaldo Failla, Maria Stella Grando, Francesco Emanuelli

**Affiliations:** 1 Research and Innovation Centre, Fondazione Edmund Mach, via E. Mach 1, 38010 San Michele all’Adige, Italy; 2 Technology Transfer Centre, Fondazione Edmund Mach, via E. Mach 1, 38010 San Michele all’Adige, Italy; 3 Department of Agricultural and Environmental Sciences, University of Milano, via Celoria 2, 20133 Milan, Italy; 4 Center Agriculture Food Environment (C3A), University of Trento, via E. Mach 1, 38010 San Michele all’Adige, Italy

**Keywords:** Grapevine, Rootstocks, Drought stress, Genome-wide association study, Candidate gene

## Abstract

**Background:**

Understanding the complexity of the vine plant’s response to water deficit represents a major challenge for sustainable winegrowing. Regulation of water use requires a coordinated action between scions and rootstocks on which cultivars are generally grafted to cope with phylloxera infestations. In this regard, a genome-wide association study (GWAS) approach was applied on an ‘ad hoc’ association mapping panel including different *Vitis* species, in order to dissect the genetic basis of transpiration-related traits and to identify genomic regions of grape rootstocks associated with drought tolerance mechanisms.

The panel was genotyped with the GrapeReSeq Illumina 20 K SNP array and SSR markers, and infrared thermography was applied to estimate stomatal conductance values during progressive water deficit.

**Results:**

In the association panel the level of genetic diversity was substantially lower for SNPs loci (0.32) than for SSR (0.87). GWAS detected 24 significant marker-trait associations along the various stages of drought-stress experiment and 13 candidate genes with a feasible role in drought response were identified.

Gene expression analysis proved that three of these genes (*VIT_*13s0019g03040, VIT*_17s0000g08960*, *VIT_18s0001g15390*) were actually induced by drought stress.

Genetic variation of VIT_17s0000g08960 coding for a raffinose synthase was further investigated by resequencing the gene of 85 individuals since a SNP located in the region (chr17_10,497,222_C_T) was significantly associated with stomatal conductance.

**Conclusions:**

Our results represent a step forward towards the dissection of genetic basis that modulate the response to water deprivation in grape rootstocks. The knowledge derived from this study may be useful to exploit genotypic and phenotypic diversity in practical applications and to assist further investigations.

**Supplementary Information:**

The online version contains supplementary material available at 10.1186/s12870-020-02739-z.

## Background

Climate change is strongly influencing human life and natural systems [1, 2], having a drastic impact on agriculture worldwide, and so viticulture must also face these new environmental conditions. Drought is the factor, among abiotic stressors, which mostly affects plant physiology [3]. Therefore, understanding the complexity of the plant’s response to water deficit poses a major challenge for researchers.

Grapevine is considered to be a relatively drought tolerant plant, thus the impact of climate change on viticulture sustainability is subject of lively debate [4–7]. Nevertheless, strategies to reduce water consumption and to improve water-use efficiency (WUE) in vines are fundamental for the future [8]. Water deficit strongly affects fruit quality and causes significant losses in crop yield. In particular, prolonged droughts could have consequences for the upcoming growing seasons [9] and may enhance susceptibility to biotic pests or pathogens [10].

Drought-stress response is the result of complex and dynamic physiological, biochemical and molecular processes at cellular and systemic levels. Water deficit leads to vegetative development [11], stomatal conductance [12, 13] and xylem hydraulic conductivity [14] reduction. Likewise, drought induces mechanisms to counteract the deleterious effects of ROS [15–17], to adjust cellular homeostasis [18] and to improve the water uptake [19–21]. The adjustment of plant water balance is also strongly influenced by phylloxera-resistant rootstocks [22], which exhibit a large variability in drought tolerance [23] and have a prominent role in regulation of stomatal conductance [24–26]. However, the genetic basis of drought response in rootstocks are generally poorly understood and only few works are available regarding whether rootstocks alter the gene expression of scions [27, 28] or whether there is an exchange of genetic material between them [29, 30].

Previous studies aimed to identify the genetic basis of drought response in grapevine [24, 31] and were conducted on biparental populations. Few Genome-wide association studies (GWAS) have been reported in grapevine yet, and interesting associations were found for fruit quality traits [32], leaf morphology [33] and domestication-related traits [34]. However, there are no reports to date of GWAS conducted to reveal the genetic control of drought response in grapevine. Furthermore, correct and accurate phenotyping plays a pivotal role in the dissection of genomic regions involved in drought tolerance [35]. In this regard, the application of chlorophyll fluorescence, near infrared (NIR) and hyperspectral imaging to assess grapevine phenotypes has become more common in recent years [36–39]. In the present study the application of infrared thermography allowed to evaluate rootstocks response to water deficit in an ‘ad hoc’ core-collection of grapes, reducing the time for phenotypic data collection, and thus allowing the screening of numerous genotypes. A GWAS approach was adopted to dissect the genomic basis of transpiration-related traits aiming to identify genetic regions involved in drought resilience potentially relevant for crop genetic improvement.

## Results

### A genetic core collection of grape rootstocks

The study was conducted on a genetic core collection constituted by 100 *Vitis* spp. accessions, listed in Table S1. A two-steps procedure was applied in order to define this restricted set of genetically highly diverse grapevine accessions as an ‘ad hoc’ association panel. Firstly, a core collection was created from *non-vinifera Vitis* species and interspecific hybrids used for fruit production, maintained at the grapevine germplasm collection of Fondazione Edmund Mach [40], to increase the allelic diversity among wild grapevines, rootstocks and hybrids accessions based on a set of 21 microsatellites. Thus, according to the M-method, 98 accessions were enough to capture the total allelic diversity (412 alleles) existing in the 231 samples analyzed. Afterwards, 41 rootstock accessions, deriving from Milano University’s breeding program, and another six additional commercial rootstocks (Paulsen 1103, Kober 5BB, Selection Oppenheim 4, Millardet et de Grasset 41B, Millardet et de Grasset 101.14 and 140 Ruggeri) were included obtaining a panel of 145 individuals. In order to have an association panel easy to manage, which adequately captures as much genetic diversity as possible with a minimum of repetitiveness, it was further reduced to 100 samples based again on the M-method. At this step the six commercial rootstocks and four out of the 41 rootstocks derived from the breeding program (M1, M2, M3, M4) were arbitrary forced to be included. The number of different alleles retained by the SSRs in the final association panel was 425.

### Genetic diversity of the core collection

The genetic diversity within the core collection was investigated by both SSR (*n* = 21) and SNP (GrapeReseq 20 K SNPs array) markers (Table 1). Regarding SNPs, after removing low quality loci, the filtered data set was made up of 16,562 SNPs. Moreover, as a consequence of the identification of missing genotypes, SNPs with a minor allele frequency (MAF) lower than 0.1 were additionally removed, remaining a final number of 7133 filtered SNPs. The average number of effective alleles was 1.51. Concerning SSR markers, 425 different alleles (A) were obtained, averaging 20.24 per locus, and allele frequencies ranged from 2.66 to 17.42, with an average of 10.07. The values of observed (H_O_ = 0.77) and expected (H_E_ = 0.87) heterozygosity were almost three times higher than those estimated for SNP markers (0.26 and 0.32, respectively). Lastly, the value of F index (inbreeding coefficient) was higher for SNPs (0.19) than for SSR markers (0.12).

**Table 1:** Summary of genetic diversity parameters within the core collection and its three subpopulations

	Sample	N	A	A _**mean**_	A_**E**_	H_**E**_	H_**O**_	uH_**E**_	F
SSR	Hybrids	30	246	11.714	5.810	0.810	0.817	0.825	−0.009
	Breeding Rootstocks	21	195	9.286	5.465	0.781	0.759	0.801	0.033
	Roostocks/Wild	49	341	16.238	8.399	0.839	0.742	0.849	0.124
	Total	100	425	20.238	10.065	0.868	0.768	0.873	0.116
SNP	Hybrids	30	14,167	1.986	1.710	0.396	0.412	0.403	−0.031
	Breeding Rootstocks	21	13,467	1.888	1.323	0.201	0.211	0.208	−0.011
	Roostocks/Wild	49	14,197	1.990	1.337	0.219	0.189	0.222	0.167
	Total	100	14,266	2.000	1.511	0.322	0.262	0.324	0.186

*N* sample size, *A* number of different alleles, *A*_*mean*_ mean number of alleles per locus, *A*_*E*_ effective number of alleles, *H*_*E*_ expected heterozygosity, *H*_*O*_ observed heterozygosity, *uH*_*E*_ unbiased expected heterozygosity, *F* fixation index (inbreeding coefficient)

The diversity parameters changed if Hybrids, Rootstocks/Wild and Rootstocks Breeding groups were considered separately. The number of alleles at the SSR loci varied from 195 in Rootstocks Breeding to 341 in Rootstocks/Wild, while for SNP markers it ranged from 13,467 in Rootstocks Breeding to 14,197 in Rootstocks/Wild. The average effective number of alleles observed among SSR loci ranged from 5.47 in Rootstocks Breeding to 8.40 in Rootstocks/Wild, whereas for SNP ones it varied from 1.32 in Rootstocks Breeding to 1.71 in Hybrids. The expected heterozygosity level estimates within groups varied from 0.78 (Rootstocks Breeding) to 0.84 (Rootstocks/Wild) for the SSRs and from 0.20 (Rootstocks Breeding) to 0.40 (Hybrids) for the SNPs. On the contrary, the subset of Rootstocks/Wild revealed the lowest values of observed heterozygosity both for SSR (0.74) and for SNP markers (0.19). Finally, the highest values of F index were detected in Rootstocks/Wild group both for the SSRs and SNPs (0.12 and 0.17, respectively) whereas the lowest values were observed in the Hybrids subset (− 0.01 at SSR loci and − 0.03 at SNP loci).

### Population structure of the core collection

**Fig. 1:**
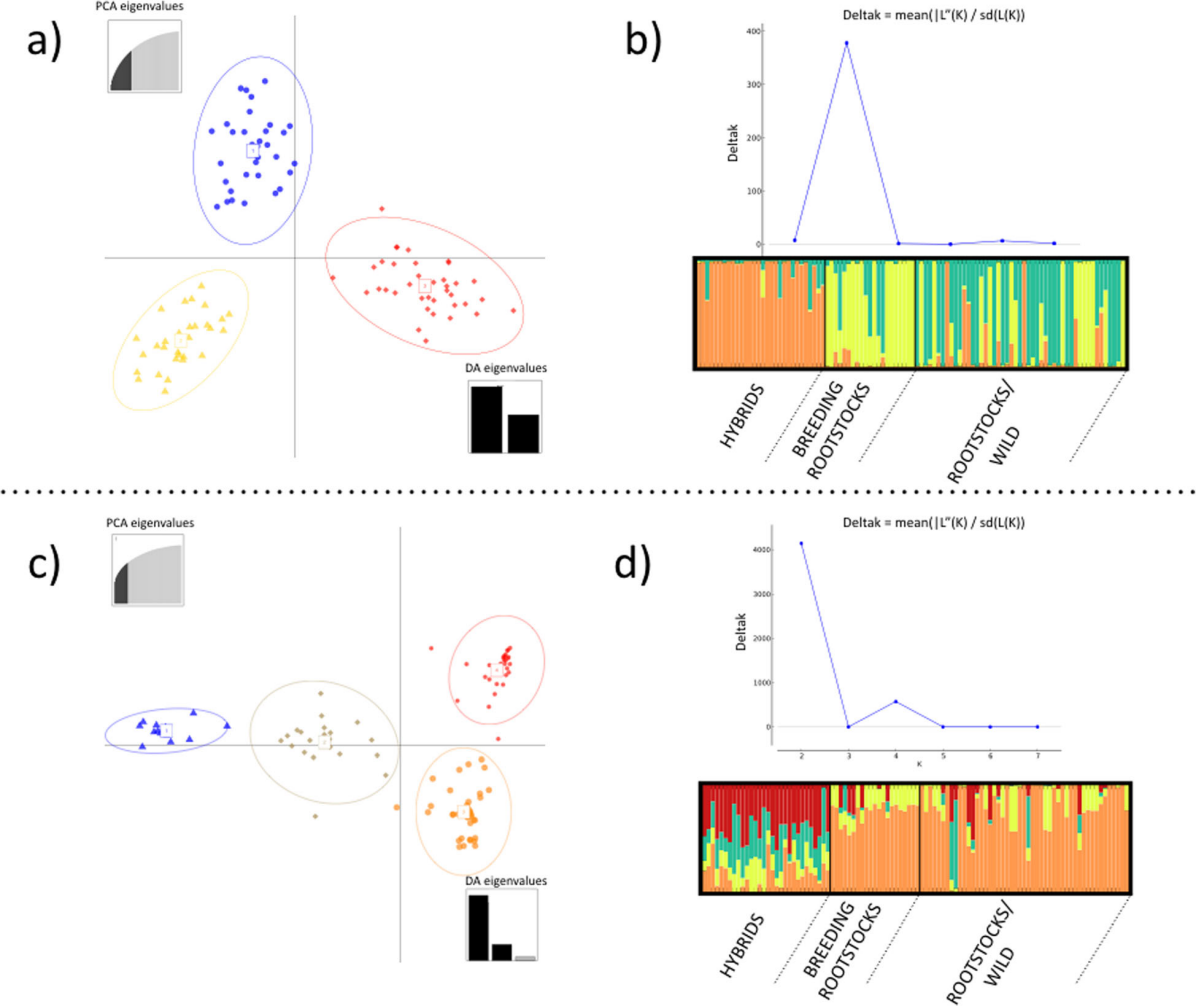
Genetic structure of the core collection. Two-dimension DAPC (Discriminant Analysis of Principal Component) scatter plot based on 21 SSRs (**a**) and on 7132 SNPs (**c**). Scatterplots represent the distribution of individuals along the first two linear discriminants. Population structure of the collection using the program STRUCTURE based on 21 SSRs (**b**) and on 7132 SNPs (**d**). Plots of mean probability of ΔK as calculated by Evanno et al. [41] to detect greatest likelihood of K. Barplots represent the average estimated membership probability of an individual to belong to a specific cluster (indicated by specific color) generated with the DISTRUCT software based on the Q-matrix consensus using the CLUMPP software

The genetic structure of the analyzed population was investigated using DAPC and STRUCTURE. The DAPC analysis identified three clusters based on SSR markers (Fig. 1a). Cluster 1 included mainly rootstocks and other *Vitis* species (59%) whereas Cluster 2 and Cluster 3 comprised most of the Breeding Rootstocks (81%) and Hybrids (100%), respectively. The model-based Bayesian clustering method in STRUCTURE software with SSR dataset (Fig. 1b) gave similar results in terms of different possible numbers of subpopulations. The ΔK method [41] assigned the highest value at K = 3, resulting in the separation of Rootstocks/Wild, Hybrids and Breeding Rootstocks in three quite distinguished groups. On the other hand, regarding the SNP dataset the same analysis showed the highest ΔK value at K = 2, dividing Hybrids from other *Vitis* species, although a minor signal of population stratification was also found at K = 4 (Fig. 1d) and, in this case, hybrids showed a high level of genetic admixture. DAPC analysis based on SNP markers also identified four clusters (Fig. 1c): Cluster 1 and Cluster 2 mainly comprised Hybrids (40 and 60% respectively), Cluster 3 included mostly Breeding Rootstocks (95%) and Cluster 4 contained a large part of Rootstocks/Wild (55%).

### Phenotypic characterization of the association panel under drought stress

**Fig. 2:**
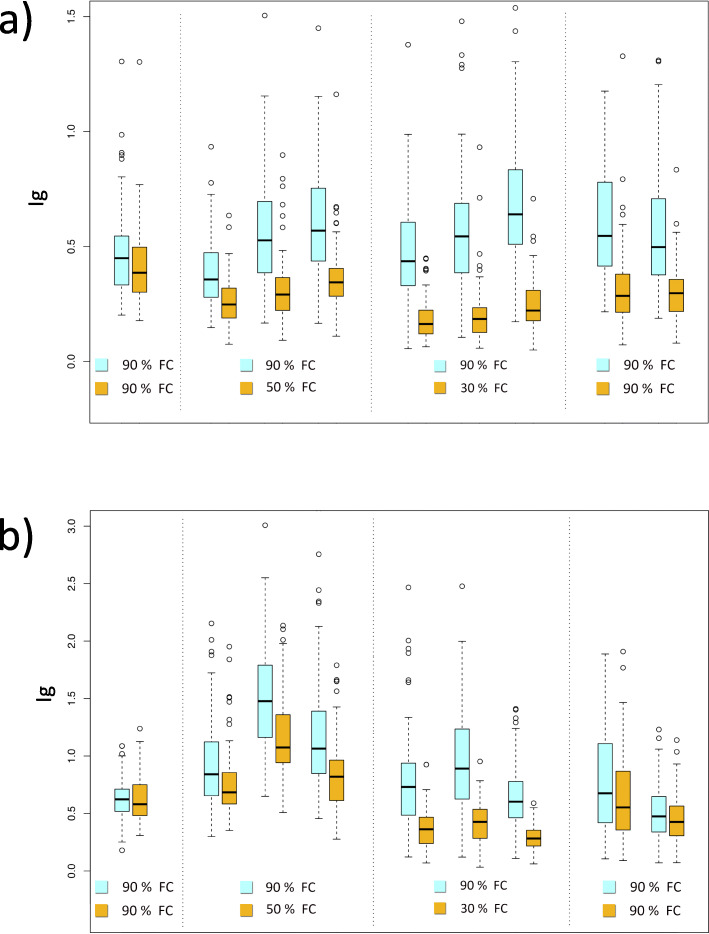
Comparison of stomatal conductance indices deduced from thermal images between well-watered (WW) (in blue) and water stressed plants (WS) (in yellow) during the water stress experiments in the first year (**a**) and in the second year (**b**)

The evaluation of the transpiration rate under drought stress in the association population was repeatedly performed in two years, in which vines of the 100 accessions were subjected to deficit irrigation (T2-T7) or were maintained in well-watered conditions for 30 days. Water stressed vines were monitored for a week at well-watered conditions before the imposition of water deficit (T1). Drought stress treatment resulted in a significant decrease of stomatal conductance (I_g_), but only in the second year experiment water-stressed plants reached transpiration values comparable with control plants after the recovery period (T8-T9) (Fig. 2). Statistically significant differences of I_g_ and Crop Water Stress Index (CWSI) were observed between control (WW) and water stressed (WS) plants in both years (Table 2).

**Table 2:** Descriptive statistics of the phenotypic data from control (WW) and water stressed plants (WS) in each year of phenotyping

Year	Time points	Treatment	FC (%)	I_**g**_		CWSI	
				Mean	SD	Mean	SD
1°	T1	WW	90	0.487	0.236	0.710	0.080
		WS	90	0.418	0.169	0.733	0.070
	T2	WW	90	0.379	0.140	0.747	0.066
		WS	50	0.264 ***	0.104	0.807 ***	0.059
	T3	WW	90	0.576	0.284	0.681	0.089
		WS	50	0.318 ***	0.153	0.785 ***	0.074
	T4	WW	90	0.623	0.295	0.655	0.088
		WS	50	0.365 ***	0.146	0.755 ***	0.067
	T5	WW	90	0.513	0.307	0.705	0.095
		WS	30	0.184 ***	0.088	0.859 ***	0.054
	T6	WW	90	0.615	0.357	0.667	0.117
		WS	30	0.205 ***	0.132	0.850 ***	0.066
	T7	WW	90	0.765	0.467	0.614	0.111
		WS	30	0.247 ***	0.117	0.821 ***	0.066
	T8	WW	90	0.625	0.317	0.660	0.103
		WS	90	0.352 ***	0.345	0.779 ***	0.097
	T9	WW	90	0.571	0.282	0.675	0.093
		WS	90	0.325 ***	0.222	0.776 ***	0.093
2°	T1	WW	90	0.627	0.183	0.639	0.068
		WS	90	0.623	0.191	0.639	0.067
	T2	WW	90	0.926	0.384	0.566	0.090
		WS	50	0.763 **	0.300	0.612 ***	0.074
	T3	WW	90	1.540	0.510	0.443	0.075
		WS	50	1.172 ***	0.343	0.503 ***	0.069
	T4	WW	90	1.220	0.623	0.518	0.089
		WS	50	0.847 ***	0.309	0.589 ***	0.079
	T5	WW	90	0.798	0.442	0.615	0.110
		WS	30	0.372 ***	0.174	0.759 ***	0.079
	T6	WW	90	0.947	0.466	0.570	0.123
		WS	30	0.410 ***	0.185	0.738 ***	0.094
	T7	WW	90	0.651	0.281	0.649	0.094
		WS	30	0.289 ***	0.108	0.795 ***	0.062
	T8	WW	90	0.782	0.438	0.621	0.125
		WS	90	0.659	0.393	0.653	0.130
	T9	WW	90	0.513	0.242	0.699	0.092
		WS	90	0.458	0.201	0.715	0.083

Asterisks denote significant differences according to Mann-Whitney U test between water-stressed (WS) and well-watered (WW) plants at the same time point. **, and *** indicate significantly different values at  *p* < 0.01, and *p* < 0.001. T1: starting point where both WW and WS plants were maintained under well-watered conditions; T2-T4: WS plants were maintained at 50% of FC (moderate deficit); T5-T7: WS plants were maintained at 30% of FC (severe deficit); T8-T9: recovery period, WS plants were rehydrated at 90% of FC

### Genome-wide association analysis

**Fig. 3:**
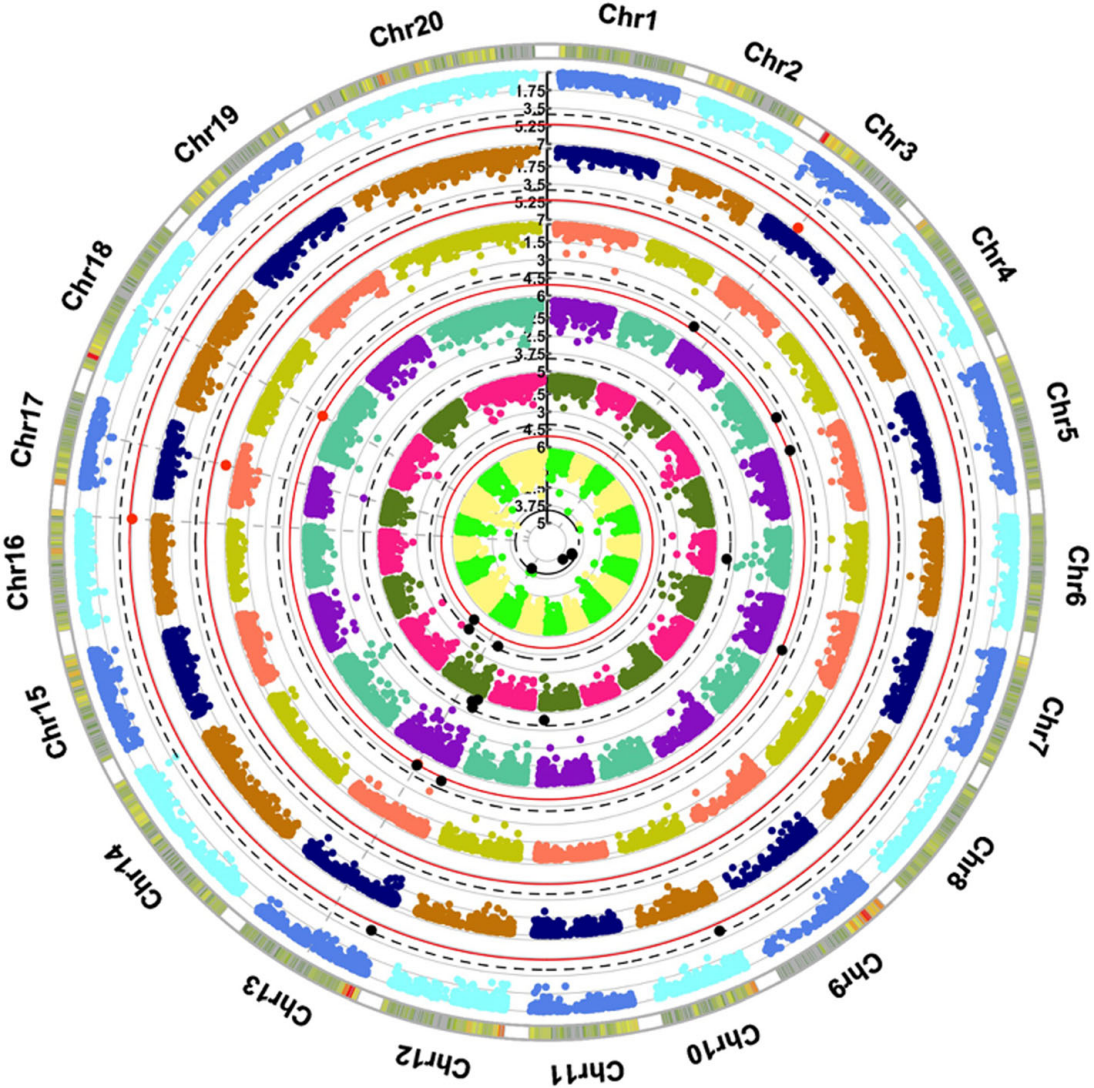
Circular Manhattan plot of association analysis between stomatal conductance (Ig) values and all SNP sites at time points T3 (aquamarin, violet), T4 (dark yellow, salmon), T5 (blue, orange), and T9 (azure, light blue) of first year experiment and time points T1 (dark green, pink) and T9 (green, yellow) of second year experiment. The red and black dots indicate respectively significant values according to the Bonferroni-corrected *p*-value and False Discovery Rate (FDR)

The GWAS was conducted for the transpiration traits related to stomatal conductance using both GLM and MLM methods. The GLM + Q was chosen as the best model based on Quantile-Quantile plots comparisons for associations found for most of the traits under investigation. The MLM + K model was instead preferred at T4 and T9 in the first year experiment. Table 3 reports twenty-four SNPs that showed significant *p*-values after multiple testing corrections. Marker-trait significant associations were identified for stomatal conductance (Ig) values at time points T3, T4, T5 and T9 in water stressed plants in the first year experiment (Figure S1). Five markers out of these 24 SNPs, identified in the first year experiment, were also significant after Bonferroni correction. The SNP chr17_10,497,222_C_T showed a significant association during severe water stress (T5), two SNPs (chr13_11,950,617_C_T, chr18_13,519,938_C_T) were statistically significant under moderate water stress (T4) and two SNPs (chr3_7,009,222_A_G and chr16_21,122,534_A_G) were significantly associated with transpiration after the recovery period (T9). Nineteen SNPs out of 24 were found significantly associated only after False Discovery Rate correction (FDR) and are, thus, identified here as suggestive associations; thirteen were detected in the first year experiment during moderate stress (T3, T4) and recovery (T9), whereas six could be found in the second year experiment at T1 and T9, that is to say, before and after (recovery) water stress, respectively. GWAS with phenotypic data collected during the second year experiment did not identify any significant association after Bonferroni correction (Figure S2). A circular Manhattan plot (Fig. 3) summarizes all the association results of both experiments.

**Table 3:** SNPs significantly associated to stomatal conductance (Ig) values

Trait	Year	SNP	Chr	Pos	***P*** value	QTL effect	R^**2**^	Multiple testing corrections
I_g_ T4	1	chr13_11,950,617_C_T	13	11,950,617	7.80E-06	A/D	0.39	BC/FDR
I_g_ T4	1	chr18_13,519,938_C_T	18	13,519,938	8.30E-06	D	0.38	BC/FDR
I_g_ T5	1	chr17_10,497,222_C_T	17	10,497,222	6.07E-07	D	0.25	BC/FDR
I_g_ T9	1	chr3_7,009,222_A_G	3	7,009,222	2.34E-07	A/D	0.50	BC/FDR
I_g_ T9	1	chr16_21,122,534_A_G	16	21,122,534	5.24E-06	A	0.56	BC/FDR
I_g_ T3	1	chr6_13,441,720_C_T	6	13,441,720	4.82E-05	A	0.22	FDR
I_g_ T3	1	chr11_18,012,075_T_C	11	18,012,075	3.28E-05	A	0.22	FDR
I_g_ T3	1	chr13_10,652,062_A_G	13	10,652,062	3.64E-04	D	0.17	FDR
I_g_ T3	1	chr13_4,177,522_C_T	13	4,177,522	2.29E-05	A	0.24	FDR
I_g_ T3	1	chr13_1,833,944_A_G	13	1,833,944	1.27E-05	A	0.18	FDR
I_g_ T4	1	chr7_17,388,970_A_G	7	17,388,970	9.99E-06	A/D	0.40	FDR
I_g_ T4	1	chr13_11,952,742_G_T	13	11,952,742	1.01E-05	A/D	0.40	FDR
I_g_ T4	1	chr5_2,431,422_C_T	5	2,431,422	1.24E-05	A/D	0.41	FDR
I_g_ T4	1	chr4_18,754,964_C_T	4	18,754,964	1.60E-05	A/D	0.37	FDR
I_g_ T4	1	chr3_235,211_C_T	3	235,211	1.60E-05	A/D	0.36	FDR
I_g_ T4	1	chr13_2,031,649_T_C	13	2,031,649	1.73E-05	A/D	0.36	FDR
I_g_ T9	1	chr10_1,989,600_G_T	10	1,989,600	4.71E-05	A/D	0.42	FDR
I_g_ T9	1	chr13_2,751,641_A_C	13	2,751,641	5.51E-05	A/D	0.40	FDR
I_g_ T1	2	chr14_3,096,968_G_T	14	3,096,968	9.96E-06	A	0.25	FDR
I_g_ T1	2	chr13_4,177,522_C_T	13	4,177,522	4.98E-05	A	0.22	FDR
I_g_ T9	2	chr7_17,388,970_A_G	7	17,388,970	3.78E-05	A/D	0.23	FDR
I_g_ T9	2	chr7_20,777,757_C_T	7	20,777,757	4.36E-05	D	0.27	FDR
I_g_ T9	2	chr9_553,031_C_T	9	553,031	2.04E-05	A/D	0.27	FDR
I_g_ T9	2	chr13_11,950,617_C_T	13	11,950,617	6.57E-05	A/D	0.23	FDR

SNPs significantly associated according to the  Bonferroni Correction (BC) and False Discovery Rate (FDR). R^2^: the proportion of phenotypic variance explained by the marker. Positions are referred to V1 annotation of the *Vitis vinifera* genome (http://www.genoscope. cns.fr)

The associated SNPs were examined to identify potential candidate genes. Firstly, it was considered whether polymorphisms would be localized in genic regions. Out of the 24 significant SNPs (Table 3) 15 were located within genes, while the remaining SNPs were in intergenic regions. For those markers located outside gene regions or in genes functionally non-annotated, the 20 kilobases surrounding them were scanned, since Linkage Disequilibrium (LD) is reported to decay rapidly in grapevine [34, 42–44]. Thirteen candidate genes, selected according to their biological functions related to water stress response or for their position, are listed in Table 4.

**Table 4:** List of candidate genes functionally annotated

Trait	Year	Candidate gene	Description	Chr	Start	Stop
I_g_ T4	1	*VIT_18s0001g15390*	Peroxidase	18	13,521,135	13,522,636
I_g_ T5	1	*VIT_17s0000g08960*	Raffinose synthase	17	10,494,444	10,498,141
I_g_ T9	1	*VIT_03s0091g00570*	Transcription factor	3	6,998,808	6,999,512
I_g_ T9	1	*VIT_16s0098g00780*	Iaa-amino acid hydrolase	16	21,120,452	21,126,524
I_g_ T9	1	*VIT_16s0098g00760*	Transcription factor	16	21,111,871	21,115,426
I_g_ T3	1	*VIT_06s0009g01570*	Serrate rna effector molecule	6	13,438,002	13,465,222
I_g_ T3	1	*VIT_11s0052g00570*	Auxin-induced protein 5NG4-like	11	18,007,469	18,008,509
I_g_ T3	1	*VIT_13s0106g00790*	Mevalonate diphosphate decarboxylase	13	10,642,954	10,652,636
I_g_ T3	1	*VIT_13s0019g03040*	Glycosyltransferase	13	4,177,111	4,179,273
I_g_ T4	1	*VIT_05s0020g00540*	β-xylosidase/α -arabinofuranosidase	5	2,435,691	2,438,632
I_g_ T9	1	*VIT_10s0003g00760*	Glutamate receptor protein	10	1,992,263	1,998,191
I_g_ T1	2	*VIT_14s0128g00480*	Eukaryotic translation initiation factor 3 subunit J	14	3,092,047	3,097,166
I_g_ T9	2	*VIT_09s0002g00810*	Peroxisomal (S)-2-hydroxy-acid oxidase GLO4	9	547,420	552,404

Positions are referred to V1 annotation of the *Vitis vinifera* genome (http://www.genoscope. cns.fr)

### Validation of GWAS results

**Fig. 4:**
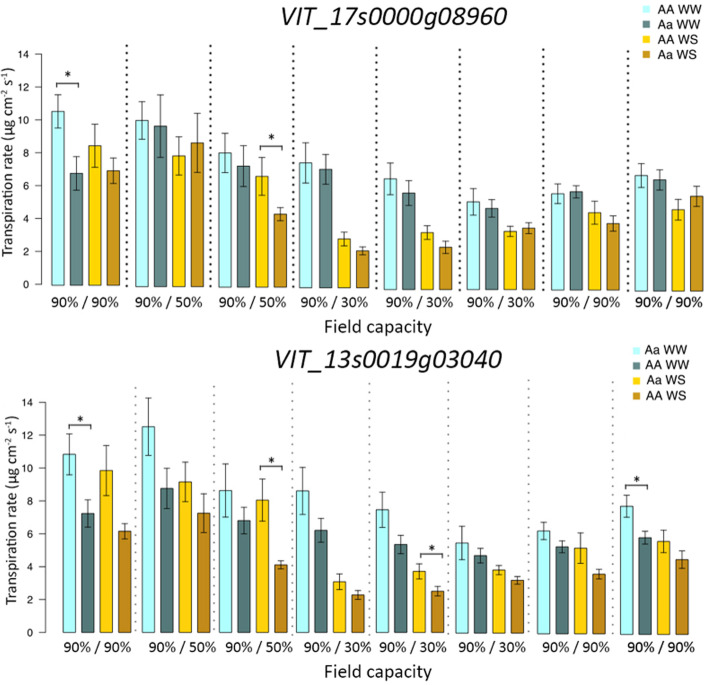
Comparison of transpiration rate between well-watered (WW) (in blue) and water stressed plants (WS) (in yellow) during the water stress experiment in the third year. Plants were divided for SNPs in the coding regions of *VIT_17s0000g08960* and *VIT_13s0019g03040*. Asterisks denote significant differences according to Mann-Whitney U test between plants on the same time point and under the same treatment at *p* < 0.05

To validate the marker-trait association found for SNPs within genes *VIT_17s0000g08960*, *VIT_16s0098g00780, VIT_13s0106g00790* and *VIT_13s0019g03040,* 16 rootstock varieties were selected for a further water stress study. Transpiration rates of WW and WS plants were measured throughout the experiment with a steady state porometer. Significant differences were detected between heterozygous and homozygous plants for SNPs in the coding regions of *VIT_17s0000g08960* and *VIT_13s0019g03040* (Fig. 4), whereas no difference was found in stomatal conductance for plants carrying different genetic variants in genes *VIT_16s0098g00780* and *VIT_13s0106g00790* (data not shown). As far as *VIT_17s0000g08960* is concerned, rootstocks heterozygous for this SNP exhibited a significant reduced transpiration rate compared with other varieties at the beginning of stress (T1) in WW plants and at moderate water stress (T3) in WS plants (Table S2, Fig. 4) although stomatal conductance values were lower also during severe stress (T4, T5). Regarding the mutation of *VIT_13s0019g03040,* significant differences between homozygous and heterozygous groups were found at moderate (T3) and at severe stress (T5) in water-stressed plants and at the beginning and end of the experiment (T1, T8) in control plants (Fig. 4, Table S2).

### Gene expression analysis of candidate genes under drought stress

The expression of candidate genes *VIT_17s0000g08960*, *VIT_18s0001g15390*, *VIT_16s0098g00780, VIT_13s0106g00790* and *VIT_13s0019g03040* under water stress was further investigated. Four rootstock genotypes (Richter 110, Riparia Gloire de Montpellier, 101.14 Millardet et de Grasset and SO4 Selection Oppenheim) were subjected to stress by water deprivation for 14 days. These genotypes were selected both to represent putatively different classes of response to water stress and based on the SNP chr17_10,497,222_C_T at the candidate gene *VIT_17s0000g08960* (Table 5).

**Table 5:** Rootstocks classification based on response to drought

Genotype	WS response class [22]	SNP chr17_10,497,222_C_T (CT overdominance effect)
SO4 (*V. riparia x V. berlandieri*)	sensitive (1) / resistant (2)	CT
101.14 (*V. riparia x V. rupestris*)	sensitive (1,2)	TT
110R (*V. rupestris x V. berlandieri*)	highly resistant (1,2)	TT
Riparia Glorie de Montpellier (*V. riparia*)	very sensitive (1,2)	CC

Response to drought in field (1) and in greenhouse (2)

**Fig. 5:**
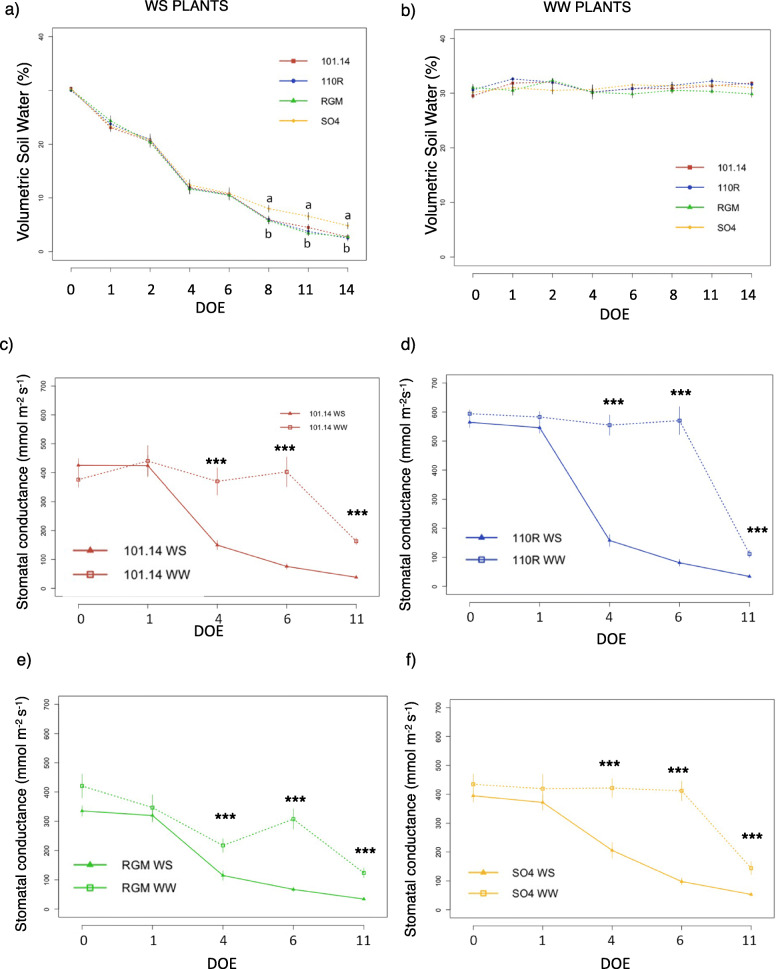
Physiological conditions of rootstocks during water stress experiment. Volumetric soil water content evaluated throughout the progression of the drought stress experiment of water stress group (**a**) and of control group (**b**). Values represent average measurements ± SE of twelve replicates (WS) and six replicates (WW). Data were analysed using one-way ANOVA with LSD *post-hoc* test, and letters indicate significant differences between genotypes on the same day at *p* < 0.05. Stomatal conductance of water stressed (WS) and well watered (WW) 101.14Mgt (**c**), 110R (**d**), RGM (**e**) and SO4 (**f**) throughout the experiment. Values represent average measurements ± SE, *n* = 12 (WW) and *n* = 24 (WS). Significant differences between treatments on the same day were tested with Mann-Whitney U test, and asterisks indicate significantly different values at *p* ≤ 0.05 (*), *p* ≤ 0.01 (**), and *p* ≤ 0.001 (***). DOE, days of experiment

Volumetric soil water content and stomatal conductance were determined throughout the experiment to monitor the stress evolution (Fig. 5). WS plants (water stress) showed a continuous decrease in soil water content with a substantial decline at four days from the beginning of the experiment. Interestingly, starting from 8 days after stopping irrigation, water content was significantly higher in SO4 compared to other genotypes. On the other hand, soil moisture in the WW control plants was maintained around 30% during the entire experimental period. Stomatal conductance, which is considered a reference parameter of plant status in response to drought, was significantly reduced by water deficit in all the rootstocks from the fourth day. Plants exhibited different degrees of tolerance after water deprivation for two weeks. Leaves of SO4 remained almost green and turgid, whereas 110R and 101.14 showed some signs of plant stress and RGM vines were considerably damaged (Figure S3).

**Fig. 6:**
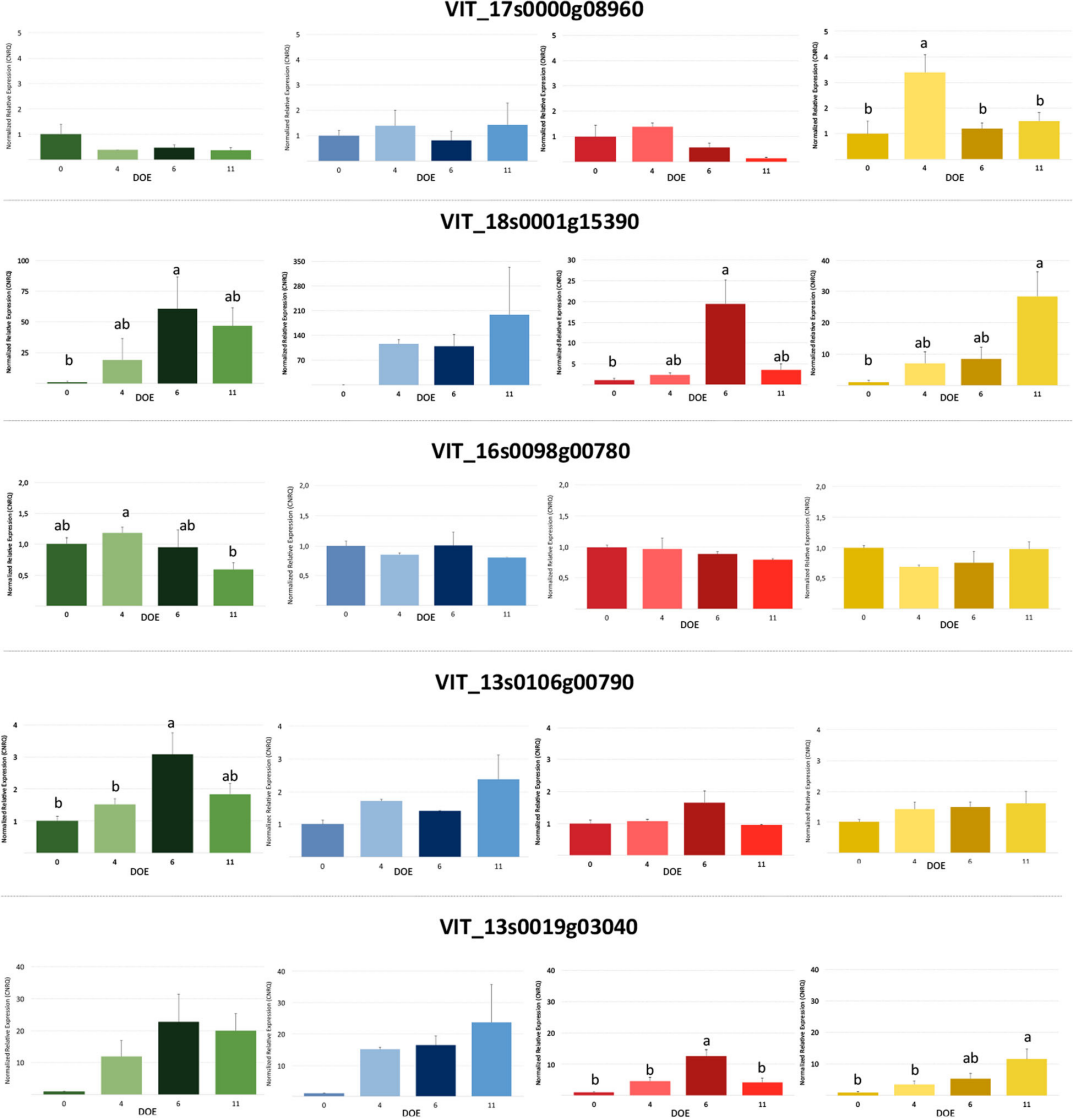
Relative expression of the 5 candidate genes assayed by quantitative real-time RT-PCR analyses in the leaves of 101.14Mgt (red), 110R (blue), RGM (green) and SO4 (yellow) sampled throughout the drought stress experiment. The y-axis indicates the folds of gene expression relative to the first day (day 0). Data are presented as means ± standard errors of three biological and two technical replicates. Data were analysed using one-way ANOVA with LSD *post-hoc* test, and letters indicate significant differences between days of experiment at p < 0.05. DOE, days of experiment

The expression of candidate genes during progressive drought stress was investigated in the leaf tissues of the four genotypes (Fig. 6). The mRNA level of *VIT_17s0000g08960* in SO4 was significantly higher after 4 days of water deprivation when plants showed first signs of stress and a notable reduction of transpiration (Fig. 5), whereas its expression decreased in the later stages of the experiment. On the other hand, the relative expression of *VIT_17s0000g08960* did not vary in response to drought in RGM, 110R and 101.14. Regarding *VIT_18s0001g15390* its transcript level was increased by water deficit, even if the peak of expression was after 6 days in RGM and 101.14 and after 11 days in 110R and SO4. A comparable time-course profile was observed for the *VIT_13s0019g03040* mRNA that was significantly modulated in the four genotypes. *VIT_13s0106g00790* was up-regulated only in RGM plants, with higher level of expression at 6 days. Finally, the relative expression of *VIT_16s0098g00780* remained basically unchanged during the experiment.

### Resequencing of candidate gene *VIT_17s0000g08960* in the association population

In order to detect a potential causative variant, the coding region of *VIT_17s0000g08960* was sequenced in 85 individuals of the core collection (Table S1). A summary of the key genetic diversity parameters observed through analysis of 2343 bp of the coding sequence is shown in Table 6.

**Table 6:** Summary statistics of VIT_17s0000g08960 protein coding region sequencing in the grape rootstocks population

Parameters	N	Synonymous changes	Non-synonymous changes
*Vitis* accessions	85		
Full-ORF cDNA	2343 bp		
Predicted protein	780 aa		
Exons	4		
Introns	3		
Number of polymorphic sites	135		
SNPs	134	65	69
INDELS	1		
Nucleotide diversity (π)	0,007	0,015	0,005
Watterson’s estimator (θ)	0,011		

*VIT_17s0000g08960* contains 4 exons as reported in the gene annotation deposited on Grape Genome Database v2.1. Resequencing allowed identification of 134 SNPs, revealing a frequency of polymorphic sites equal to one SNP every 17 bp. Only one INDEL was found in exonic regions. The nucleotide diversity (π = 0,007) and the number of segregating sites (θ = 0,011) provided an estimate of the genetic variation at the nucleotide level. Synonymous sites, on the other hand, showed a nucleotide diversity value (0.015) much higher than non-synonymous sites (0.005). In addition, genetic variation level estimated by dividing *Vitis* accessions into two subsets (Rootstocks and Hybrids) is reported in Table S3. The Rootstocks subset exhibited greater rate of polymorphisms (one SNP every 21 bp) and lower nucleotide diversity (π = 0,006, θ = 0,011) than the Hybrids subgroup (one every 33 bp, π = 0,007, θ = 0,007). Neutrality tests were estimated using two values, Tajima’s D value and Fu and Li’s F value. Both tests indicated that the polymorphisms did not show any significant deviation from neutrality neither in the whole dataset nor in the subsets of Rootstocks and Hybrids. The impact of non-synonymous substitutions on the biological function of the protein was predicted for all 69 mutations detected. Seventeen showed a PROVEAN score below − 2.5, which indicates a probable structural alteration of protein (Table S4). Additionally, some of these deleterious mutations occur in a significant proportion of the rootstock population.

## Discussion

### The genetic core collection of grape rootstocks

The existing grape germplasms are valuable genetic resources that could be examined for seeking phenotypic variations in drought tolerance mechanisms. Constructing a genetic core collection has proved to be an adequate strategy to obtain an optimal number of rootstock genotypes which captures all the most frequent alleles of a large germplasm, which is in agreement with previous studies [40, 42, 45]. Moreover, the use of a genetic core collection for marker-trait association studies was applied in several plant species with excellent results [42, 46–48]. Our results showed that a relatively few accessions were required to represent the whole genetic diversity with the minimum redundancy, probably due to the high heterozygosity of *Vitis* species [49]. Similar outcomes have been reported in *Malus* [50], which exhibits high levels of heterozygosity as well, whereas more individuals were needed in *M. truncatula* [51] that is characterized by lower allelic heterozygosity.

A considerable level of genetic diversity within the core collection has been confirmed by the analysis with microsatellites and SNP markers and both proved to be highly informative. Conversely, the level of heterozygosity estimated by SNPs was substantially lower compared to that obtained with SSRs, as expected, since markers of bi-allelic nature have a lower discrimination power and detect a smaller proportion of rare alleles in a population [52, 53]. The slight reduction of Ho in comparison with H_E_ may be the result of inbreeding events on the population under investigation, as noted in previous studies [40, 54, 55], and the low F values are also attributable to the high heterozygosity of grapevine. Furthermore, an overall reduction of genetic diversity has been observed within the Breeding Rootstocks pool compared to the Rootstocks/Wild and Hybrids panels, because they were obtained through a breeding selection based on few progenitors.

Regarding the genetic structure of the core collection, Hybrids were grouped clearly in a distinct cluster separated from Breeding Rootstocks and Rootstocks/Wild. This outcome was predictable because hybrids were obtained by crossing American *Vitis* species with cultivated grapevines carrying both phylloxera resistance and a significant percentage of *V. vinifera* in their pedigree [56].

### Phenotyping of drought stress response

Grapevine WUE under droughts is strongly influenced by plant transpiration rate, which can be, therefore, considered a potential target for its improvement [57]. Thermal infrared imaging was confirmed as a very suitable tool for the estimation of stomatal conductance and to study the genotypic variability related to transpiration. During the three experimental years, rootstocks exhibited significantly higher canopy temperatures in comparison with their controls when subjected to water stress, reflecting their water status. Moreover, it was demonstrated that Ig and CWSI parameters, deduced from thermal images, were significantly correlated with water stress indicators, such as leaf water potential (ΨL), non-photochemical quenching (NPQ) or efficiency of light use by the photosystem II (PSII) [58]. The timing of measurements is critical to ensure satisfying phenotyping results and this approach allowed a fast assessment of the transpiration rate in the whole rootstock population (600 vines) in the same day and during a specific time window to limit environmental influence, which would have been impossible with a porometer. In fact, since the initial development of the thermography method by Blum et al. [59], water status of different kind of crops has been widely studied with excellent results in diverse research works, including grapevine [38, 39, 60–62]. This experiment demonstrated the effectiveness of thermal imaging in detecting genome wide-associations overall. Moreover, the analysis of drought response on a subset of the population using direct stomatal conductance measurements proved the consistency of these outcomes. Nevertheless, it is essential to investigate other morphological characteristics, such as vegetative development or root architecture, and evaluate physiological aspects of rootstock-scion interaction in both pot and field experiments, in order to acquire a full knowledge of the plant physiological response.

### Genome-wide association analysis

GWAS studies are currently a valuable approach to understand the genetic basis of complex traits [63], particularly for those with polygenic inheritance, such as drought tolerance, although these analyses are not widely carried out in grapevine [32–34, 64–66]. According to Nicolas et al. [43] the ideal association panel for GWAS in grapevine should combine limited relatedness with minimal structure. The panel designed for this study was composed by hybrids, wild non-*vinifera* accessions and rootstock varieties (Table S1) that included in their pedigree the main American *Vitis* species, such as *V. riparia*, *V. berlandieri* and *V. rupestris*. Therefore, it ensures a large genetic variability and, additionally, exhibits unexplored variations for biotic and abiotic stresses resilience [67, 68]. However, GWAS analysis identified only five SNPs which passed the Bonferroni significance threshold associated with the studied phenotypic traits. On the other hand, nineteen marker-trait associations were detected using FDR’s less conservative approach. The decrease of statistical power could be caused by the rapid decay of LD in grape [43, 66, 69] that might require a large number of SNPs to evenly cover the genomic region. The GrapeReseq 20 K SNPs array proved to be an adequate tool to detect significant genotype-phenotype associations in this study. This chip includes probes targeting variations discovered within 47 wild and cultivated genotypes of *V. vinifera*, but also 4978 SNPs identified in 18 accessions of other six *Vitis* species. Therefore, it was appropriate for genotyping the core collection, which contains several different *Vitis* genotypes. Moreover, the SNP markers on array were selected based on their level of heterozygosity and evenly distributed along chromosomes. This array, in fact, has allowed detection of QTLs for vegetative and reproductive traits [Houel et al. 2015] and a great estimation of genetic diversity in grapevine germplasms [33, 53, 64]. On the other hand, a greater number of SNP markers could have been obtained through either the Restriction-site associated DNA sequencing (RADSeq) or the Genotyping by Sequencing (GBS) approach. Maximizing the number of SNPs may indeed increase the likelihood of finding significant associations with the phenotype. However, this strategy requires a more accurate filtering of the SNPs discovered and needs to be previously optimized for the different genotypes under investigation. In the present study the plants under investigation are either hybrids or accessions from different *Vitis* species and it is possible that they may differ in terms of the presence of restriction enzymes sites in highly repetitive DNA regions. Furthermore, drought tolerance is a trait with a complex polygenic determinism and with a strong environmental interaction and, hence, a marker-trait association analysis will probably require highly precise phenotypic data, and an experimental panel including more individuals and replicates, in order to detect minor effect QTLs.

The prominent role of rootstocks in regulating scion stomatal conductance under water deficit has been demonstrated in different studies [25, 26, 70, 71], although the genetic determinism involved in the stomatal regulation has been scarcely investigated. Marguerit et al. [24] identified, through a QTL analysis, genetic regions in rootstocks linked to the transpiration control of scions by evaluating drought response of a single scion genotype grafted on 138 individuals from a *V. vinifera* cv. Cabernet Sauvignon × *V. riparia* cv. Gloire cross. Later, Coupel-Ledru et al. [31] dissected the genetic basis of stomatal sensitivity between iso- and aniso-hydric grapevines in a progeny (*Vitis vinifera* L. cvs. Grenache × Syrah) again with a QTL approach. So far, these remain the only studies focused on identifying the genetic regions responsible for stomatal control under water stress.

The association mapping approach adopted in this study detected significant genotype- phenotype associations during the various stages of drought stress of the experiment. GWAS results of the second year, however, were not consistent with those obtained during the first year. This fact highlights the need to cope with challenges of plant phenotyping for drought tolerance, which may be influenced by multiple abiotic stress conditions, such as excessive heat [35, 72]. Indeed, despite the experiments were conducted in a tunnel- greenhouse, external conditions were slightly different likely having an impact on inside temperature. Differences were also noticed regarding the transpiration response. Environmental conditions reduced the stomatal conductance of control plants in the first year and water-stressed plants exhibited a transpiration rate close to zero at 30% of FC, showing only a partial recovering after rehydration. Nevertheless, phenotypic data collected in a third year on a subset of the population with a porometer, which measures more accurately the stomatal conductance, confirmed some of the associations.

Previous genetic studies of the grapevine transpiration under drought [24, 31] reported a comprehensive characterization of the population over the course of the water treatment, even though the low density of markers limited the resolution of QTL confidence intervals, which included large chromosomic regions. However, the large amount of significantly associated SNPs identified in this study co-localized in those QTL regions, even indicating more restricted positions. Therefore, the application of an integrated strategy combining QTL mapping and GWAS analysis seems a valid approach to dissect complex traits, such as drought stress response.

### Potential candidate genes for drought tolerance

The significantly associated SNP chr17_10,497,222_C_T (*p* < 0.0001) was identified under severe water deficit conditions in the first year experiment. Moreover, other association signals for the same marker, which did not exhibit significant *p*-values after multiple testing corrections, were found in the first year (at 50% of FC) and in the second year (at recovery stage). Additionally, the association of the SNP with a different rate of transpiration under drought was validated in a small group of rootstock varieties in a third year experiment. Indeed, genotypes with heterozygous SNP (CT) exhibited a significant reduction of stomatal conductance compared with genotypes carrying homozygous SNP (CC or TT) at 50% of FC. The SNP chr17_10,497,222_C_T is located in the coding region of *VIT_17s0000g08960*, which codes for a raffinose synthase. The raffinose family of oligosaccharides (RFOs) has a fundamental role in protecting plants against abiotic stresses [73]. These proteins confer tolerance against drought stress acting as signaling compounds through the phloem, and as storage of additional energy resources. In addition, they have a ROS scavenging function and stabilize cellular membranes and photosynthetic apparatus. The accumulation of these carbohydrates also improved the water stress tolerance in several plants, such as *Arabidopsis thaliana* [74–76], *Medicago sativa* [77], *Xerophyta viscosa* [78], *Zea mays* [79], *Coffea* [80] and *Malus domestica* [81]. Grapevines subjected to drought generally show an overall reduction of sugars [82], probably due to a decreased carbon fixation, except for galactinol and raffinose, which accumulate upon water deficit conditions [17], suggesting, therefore, that their biosynthesis is strictly related to stress. Furthermore, the concentration of osmolytes like raffinose in guard cells has a role in the regulation of stomata aperture [83, 84]. The involvement of *VIT_17s0000g08960* in drought response mechanisms, including ABA-mediated signalling, is confirmed by transcriptomic studies in grapevine. It was differentially modulated in leaves of isohydric and anisohydric varieties under water deficit conditions [85] and it was up regulated in Merlot grapevine leaves subjected to drought [17], in transgenic grape cells overexpressing *VvABF2* [86] and in berries after ABA treatment [87]. Phylogenetic analysis of the protein codified by *VIT_17s0000g08960* has also demonstrated that it is closely related to stress-inducible protein raffinose synthase 5 (RS5) of Arabidopsis, that has proved to be the solely responsible for raffinose accumulation in leaves under water stress [88]. The role of *VIT_17s0000g08960* in drought stress response was also supported by the in silico analysis of its promoter, which exhibited a consistent enrichment for major ABA-responsive elements (ABRE) and dehydration-responsive element binding (DREB) motifs (ACGTG, RYACGTGGYR, YACGTGGC, ACGTGKC, ACCGAC) [89–93]. Accordingly, the *VIT_17s0000g08960* coding region has been sequenced in 85 rootstock genotypes in order to detect a potential causative variant. Its nucleotide diversity (π = 0,007) is higher than the average values observed in grapevine gene regions reported in literature (π = 0,0040-0,0051) [42, 94–96], which is consistent to the complex nature of the highly diverse association panel that includes different *Vitis* species and hybrids and thereby presenting a large genetic variability. Interspecific hybrids, which all include *V. vinifera* in their pedigrees, showed a lower frequency of polymorphic sites compared with other rootstock genotypes. On the other hand, if mutations in the non-coding portions of the genome are considered, the genetic diversity in grapevine is substantially higher both in wild and cultivated varieties ranging from π = 0,015 and π = 0,014, respectively [97]. Despite a recently published whole-genome resequencing of 472 *Vitis* accessions revised downwards these estimates, nucleotide diversity values of π = 0,0035 for wild and π = 0,0055 for domesticated cultivars were reported [49]. Unfortunately, none of the non-synonymous changes of *VIT_17s0000g08960* coding region proved to be in LD with the associated variant identified in GWAS. Thus, the putative causative mutation in LD with the significant synonymous SNP could be located in genomic regions that have not been sequenced; cis-regulatory sequences can be localized in intragenic (introns) or intergenic (promoter and enhancer) regions closely surrounding the gene and need to be further investigated.

In the GWAS experiment SNP chr17_10,497,222_C_T was significantly associated with stomatal closure in drought stress conditions with an overdominance effect, heterozygous (CT) genotypes showed lower stomatal conductance in comparison with homozygous genotypes (CC or TT). In this respect, commercial rootstocks (representative of the three genotypic classes) were deeply characterized under drought in a pot stress experiment. Interestingly, as soil water content decreased SO4 vines proved to be the more able to preserve soil moisture. In accordance with our results, Tramontini et al. [25] reported that different grapevine genotypes grafted on SO4, grown under water-limiting conditions in small pots, preserved the soil water in a more efficient way compared with the same varieties grafted on high tolerant rootstock, 140 Ruggeri. The analysis of the *VIT_17s0000g08960* transcripts during water deficit revealed that this gene was modulated only in SO4 vines. A significantly higher expression was detected after four days, when plants start to perceive the symptoms of stress, indicating that it might be implicated in early response to drought stress. Therefore, the potential causative mutation could have a role in the transcriptional regulation.

Among the other significantly associated markers, SNP chr18_13,519,938_C_T is positioned within the promoter region of another drought responsive gene, *VIT_18s0001g15390*, which encodes a peroxidase protein. Peroxidases are antioxidant enzymes that prevent excessive damages caused by ROS accumulation and their concentrations are highly modulated under abiotic stresses [98, 99]. Moreover, its expression profile during drought was characterized by a progressive increase of transcripts throughout the experiment in all the four rootstocks genotypes, which supports a prominent role in the stress response. The other three statistically significant polymorphisms after Bonferroni adjustment, chr3_7,009,222_A_G, chr16_21,122,534_A_G and chr13_11,950,617_C_T, map near a TF involved in transcription initiation, in the intronic region of an iaa-amino acid hydrolase and in a non-annotated gene prediction, respectively. Since these genes could not be considered directly related to water stress response, surrounding genomic regions were scanned without finding credible candidate genes. However, these regions would deserve much more in-depth analysis because candidate gene approach could be limiting and may exclude non-coding regions actually associated with the phenotype (promoters, enhancers, silencers, etc.) [100]. The Bonferroni correction test is the most applied for assessing the threshold value of associations. Nonetheless, it is often too conservative and some signals may not pass its stringent criteria. Thus, SNPs suggested based on FDR were also considered to detect other marker-trait association. All the identified markers were found only during one stage of stress, except chr13_4,177,522_C_T. This SNP, located in the coding region of a glucosyltransferase protein (VIT_13s0019g03040), was found significant under both moderate water deficit and well-watered condition. Several recent studies in model plant species also suggest the involvement of glycosyltransferases in abiotic stress adaptation [101–103]. Furthermore, we observed that *VIT_13s0019g03040* expression increases along drought experiment in the studied rootstock varieties, so it might play a key role in drought response mechanisms. Lastly, marker chr13_10,652,062_A_G was found associated in plants under moderate drought stress and it is positioned in the coding region of mevalonate diphosphate decarboxylase (MVD) (VIT_13s0106g00790). This is a limiting enzyme of mevalonate isoprenoid pathway [104] responsible for the formation of sterols, which play an essential role in maintaining membranes structure and in preventing oxidative stress damages [105].

## Conclusions

Understanding the genetic basis of grapevine drought stress response is crucial in the management of vineyards and in the breeding of new varieties in a changing climate. In the present research, some genetic regions related to the control of transpiration potentially involved in drought resilience and relevant for crop improvement were detected with a GWAS approach. The application of infrared thermography allowed evaluating the grapevine rootstocks response to water deficit reducing the time for collecting phenotypic data, and, thus, allowing the screening of numerous genotypes. Significant marker-trait associations were detected, despite the complexity of the trait under investigation and its polygenic inheritance. Additional studies on commercial rootstocks enabled us to point out several candidate genes (*VIT_*13s0019g03040, VIT*_17s0000g08960*, *VIT_18s0001g15390*) presumably implicated in response to water deficit, providing valuable information on important tolerance traits. These results highlighted a relevant role of a raffinose synthase, belonging to a family of oligosaccharides well known for protecting plants against abiotic stresses.

## Methods

### Plant material and construction of genetic core collection

The association population consisted of one hundred non-*vinifera* genotypes (*Vitis* spp*.)* representing the genetic diversity of two more extensive germplasm collections maintained by Fondazione Edmund Mach (ITA362) [40] and University of Milan (ITA427). The material consisted of interspecific hybrids used for fruit production (Hybrids), rootstock varieties including wild *non-vinifera Vitis* species (Rootstocks/Wild) and rootstocks selected in a breeding program (Rootstocks Breeding). The MSTRAT software, which implements The Maximization (M) method [106, 107], was applied to construct this core collection, by performing 200 iterations per MSTRAT run and 100 repetitions for core sampling. Putative core collections with equal allelic richness were ordered according to Nei’s diversity index [108]. The final core collection included the accessions that were more frequent in the 100 replicates.

### SNP genotyping, genetic diversity and genetic structure of the population

DNA was isolated from leaves of rootstock genotypes with the DNeasy® Plant Mini Kit (QIAGEN, Hilden, Germany). DNA quality was assessed using both agarose gel electrophoresis and the NanoDrop ND-8000 spectrophotometer (NanoDrop Technologies, Wilmington, DE, USA).

The commercial GrapeReseq 20 K SNPs array was used to genotype the core collection with the Infinium technology following the manufacturer’s instructions (Illumina, Inc., San Diego, CA, USA). The raw SNP data generated were scored and filtered according to Marrano et al. [34].

Genetic variability within and among groups was measured both for SSR and SNP loci. The mean number of alleles per locus (A), the number of effective alleles (A_E_, [109]), levels of observed (H_O_) and expected (H_E_) heterozygosity [110] and the fixation index (F, inbreeding coefficient [111]) were calculated using GenAlex 6.502 [112].

The genetic structure of the association population was analyzed with STRUCTURE software v2.3.2 [113], which uses a variational Bayesian framework for approximate inference of subpopulations [114]. Ten independent runs for K values ranging from 1 to 7 were performed with the following parameters set (burn-in length/iterations) 500,000/750,000 and 10,000/100,000 for SSR and SNP data, respectively. The admixture model was applied with no prior population information. Estimation of the most probable K value was obtained running the algorithm for multiple choices of K and visualizing the marginal likelihood and ΔK [41] of the data over ten runs using STRUCTURE HARVESTER [115]. The optimal alignment of runs was analyzed with CLUMPP v1.1.2 [116]. Final results were visualized with the software DISTRUCT v1.1 [117].

A Discriminant Analysis of Principal Components (DAPC) [118] was performed to identify genetic clusters using the package *adegenet* of R software. The number of axes considered in the Principal Component Analysis (PCA) was determined with cross-validation (CV) function implemented in p*oppr* package of R software [119].

### Water stress experiment conditions

Six replicates of each grape genotype included in the association mapping panel were grown in a 5-L pot filled with a substrate composed of sandy loam soil and peat (4:1 in volume) under partially controlled climate conditions. Soil water content (SWC) was determined by the gravimetric method, from the difference in weight between the wet and the dry soil [120]. Two irrigation treatments were established. Three replicates were irrigated maintaining the 90% of SWC (well-watered plants, WW) and 3 replicates (water stressed plants, WS) were subjected to a gradual drought stress: a moderate stable water deficit (5o% of SWC for 7 days), followed by a severe stable water deficit (30% of SWC for 7 days) and a recovery period (90% for 5 days). This experiment was repeated for three years: 2012 (1° year), 2013 (2° year) and for a subset population in 2014 (3° year).

One-year-old potted (9 L) rooted cuttings of three selected rootstock varieties (101.14, SO4, RGM) were further grown and evaluated in a tunnel- greenhouse. Twelve replicates of each rootstock genotype were subjected to water stress by completely suspending irrigation for 15 days (WS), while 6 replicates were maintained at about 90% of maximum water availability (WW). The growing medium was composed of a sand-peat mixture (1:1 in volume) with a field capacity of 35% [(vol water/vol soil) × 100]. The volumetric soil moisture content per pot was monitored with a ML3 ThetaProbe Soil Moisture Sensor (Delta-T Devices, London, UK). The pot surface was covered with a plastic film to avoid soil water evaporation. The experimental plan was completely randomized.

### Thermal indices and stomatal conductance estimation

The physiological response to drought was evaluated over 30 days. To evaluate the effect of water stress thermal images of the grape leaf canopies were elaborated using the software InfReC Analyzer (NS9500LT) (Nippon Avionics Co., Yokohama, Japan). Stomatal conductance was estimated from two different thermal indices: crop water stress index (CSWI) (Eq. 1) [121] and thermal index (Ig) (Eq. 2) [122].

1 $$ CWSI=\frac{T_{canopy}-{T}_{wet}}{T_{dry}-{T}_{wet}} $$

2 $$ IG=\frac{T_{dry}-{T}_{canopy}}{T_{canopy}-{T}_{wet}} $$

where T_canopy_ (°C) was the temperature deduced from the thermal images of six sun-exposed mature leaves per vine, T_dry_ (°C) and T_wet_ (°C) were the temperatures detected on the cardboard “reference surfaces”. Stomatal conductance (g_s_) and transpiration were measured with a steady state porometer (Licor Li-1600) in the third experimental year.

### GWAS analysis

Genotype-phenotype associations were tested considering the average value of each trait for each year separately. When phenotype scores were not normally distributed they were transformed using the logarithm function. Three different models were tested using TASSEL v.5.2 [123]. The first model applied was the General Linear Model (GLM), which considers the population structure calculated with principal component analysis (PCA) as a cofactor. The following matrix notation describes the GLM model:

3 $$ {y}_i=\mu +{x}_i\beta + Q\nu +\varepsilon $$

where y_i_ is the phenotypic value of i^th^ sample, μ is the model intercept, β is a vector of SNP effects, ν is a vector of population effect and ε is a vector of residual effects, Q is the matrix from STRUCTURE that considers the individual probabilities to be associated to a subpopulation. The second model employed was the Mixed Linear model (MLM), which extends eq. (3) taking also into account a kinship matrix (K) to estimate the degree of genetic covariance between pairs of individuals [124]. The method of Endelman and Jannink [125] was applied to determine a centered identical-by-state K. The third model (Q + K model) including both a fixed effect as the population structure matrix (Q) and a random effect as the kinship matrix (K). The marker trait association was evaluated by plotting quantile-quantile (Q-Q) plot. *P*-values adjustment for multiple testing was adopted: in addition to the Bonferroni-corrected critical *p*-values, q-values were also calculated based on their corresponding p-values to identify significant associations between a trait and the SNPs. The q-value is a measure of significance in terms of False Discovery Rate (FDR) [126] that limits the false positive results while offering a more liberal criterion than Bonferroni correction factor. A q value of 0.1 was used as significant association threshold [127]. GWAS results were visualized with Manhattan plots that were yielded from the *qqman* and *CMplot* packages of R software [128]. Genomic regions closest to the markers significantly associated with phenotypes were explored to identify candidate genes. Taking into account the extent of Linkage Disequilibrium (LD), a window of 10 kb upstream and downstream from associated loci was considered on the grape genome assembly v.2.1, hosted on http://genomes.cribi.unipd.it/grape [129].

### *VIT_17s0000g08960* gene resequencing and genetic variation analysis

Gene-specific primers were designed using Primer 3 software [130] based on the genomic sequence of *V. vinifera* gene annotation v2.1. An assembled contiguous sequence of 3678 bp of the *VIT_17s0000g08960* locus was resequenced with primers listed in Table S5 according to methods previously described [131].

The estimation and frequency of polymorphisms were defined using the DnaSP software [132], based on the SNPs and INDELs detected in *VIT_17s0000g08960* coding region. Nucleotide diversity was estimated as π [133]. The neutral mutation parameter θ [134] was estimated from the total amount of mutations. The hypothesis of neutral polymorphisms was tested using Tajima’s D [135] and Fu and Li’s D [136] tests. Prediction of tolerability of amino acid substitution at all positions was calculated with the software tool PROVEAN (Protein Variation Effect Analyzer) [137].

### Real-time qPCR

Total RNA was isolated from grape leaves using the Spectrum™ Plant Total RNA Kit (Sigma Aldrich, St. Louis, MO, USA). DNase treatment was performed using the Dnase I (Qiagen, Valencia, CA, USA) during the RNA extraction. RNA samples were quantified with the spectrophotometer NanoDrop ND-8000 (NanoDrop Technologies, Wilmington, DE, USA) and their integrity was checked by agarose gel electrophoresis. cDNA was synthesized with the SuperScript® III Reverse Transcriptase (Invitrogen, Carlsbad, CA, USA). Analysis of candidate genes expression was carried out using LightCycler® instrument and the related LightCycler® software (Roche Diagnostics, Basel, Switzerland). All Real-Time PCR reactions were performed using LightCycler® 480 SYBR Green I Master Mix (Roche Diagnostics, Basel, Switzerland) in 20 μl reactions according to to manufacturer’s instructions by using primers listed in Table S6. Three independent biological replicates for each time point were analyzed. Gene expression levels were assessed with qbasePLUS software (Biogazelle, Zwijnaarde, Belgium [138]) and normalized by the reference genes Actin and Glyceraldehyde-3-phosphate dehydrogenase.

### Statistical analyses

Statistical analyses were performed using R packages ‘stats’, ‘agricolae’ and ‘companion’ v3.5.1 (R Core Team, 2013). Different tests were applied for mean comparisons. Parametric Student’s t-test or one-way ANOVA were used to compare normally distributed data with equal variances. Non-parametric Mann-Whitney U test and one-way Kruskal–Wallis test were applied when the assumptions of normality or homogeneity of variances were violated.

## Supplementary Information

**Additional file 1.**

**Additional file 2.**

**Additional file 3.**

**Additional file 4.**

**Additional file 5.**

**Additional file 6.**

**Additional file 7.**

**Additional file 8.**

**Additional file 9.**

## Data Availability

All data generated during this study are included within the article and in its supplementary information files or are available from the corresponding author on reasonable request. Nucleotide sequences of *VIT_17s0000g08960* can be found in the GenBank data libraries under the accession numbers: MW066760, MW066761, MW066762, MW066763, MW066764, MW066765, MW066766, MW066767, MW066768, MW066769, MW066770, MW066771, MW066772, MW066773, MW066774, MW066775, MW066776, MW066777, MW066778, MW066779, MW066780, MW066781, MW066782, MW066783, MW066784, MW066785, MW066786, MW066787, MW066788, MW066789, MW066790, MW066791, MW066792, MW066793, MW066794, MW066795, MW066796, MW066797, MW066798, MW066799, MW066800, MW066801, MW066802, MW066803, MW066804, MW066805, MW066806, MW066807, MW066808, MW066809, MW066810, MW066811, MW066812, MW066813, MW066814, MW066815, MW066816, MW066817, MW066818, MW066819, MW066820, MW066821, MW066822, MW066823, MW066824, MW066825, MW066826, MW066827, MW066828, MW066829, MW066830, MW066831, MW066832, MW066833, MW066834, MW066835, MW066836, MW066837, MW066838, MW066839, MW066840, MW066841, MW066842, MW066843, MW066844.

## Abbreviations

GWAS: Genome-wide association study
WUE: Water-use efficiency
NIR: Near infrared
MAF: Minor allele frequency
SSR: Simple sequence repeat
SNP: Single-nucleotide polymorphism
CSWI: Crop water stress index
WS: Water stress
FDR: False discovery rate
LD: Linkage disequilibrium
110R: 110 Richter
SO4: Selection Oppenheim 4
RGM: Riparia Gloire de Montpellier
101.14: 101.14 Millardet et de Grasset
WW: Well-watered
NPQ: Non-photochemical quenching
QTL: Quantitative trait locus
FC: Field capacity
RFO: Raffinose family of oligosaccharides
ABA: Abscisic acid
ABRE: ABA-responsive elements
DREB: Dehydration-responsive element binding
MVD: Mevalonate diphosphate decarboxylase
DAPC: Discriminant analysis of principal components
SWC: Soil water content
GLM: General linear model
MLM: Mixed linear model
