# Supplementary material for: Candidate genes and SNPs associated with stomatal conductance under drought stress in Vitis
Source: BMC Plant Biol. 2021 Jan 6;21:7. doi: 10.1186/s12870-020-02739-z (PMC7789618; doi:10.1186/s12870-020-02739-z)
Supplement: Supplementary file 2 — Additional file 2. [file 12870_2020_2739_MOESM2_ESM.pdf]

Tab. S2 Descriptive statistics of transpiration of plants divided for SNPs in the coding regions of *VIT_17s0000g08960* and *VIT_13s0019g03040*. Asterisks denote significant differences according to Mann-Whitney U test between plants on the same time point and under the same treatment at $p < 0.05$.

Time points	Treatment	FC (%)	SNP chr17_10497222_C_T	Number individuals	Stomatal conductance	
					Mean	SD
T1	WW	90	TT/CC	10	10,58	3,19
			CT	6	6,81 *	2,50
	WS	90	TT/CC	10	8,49	4,15
			CT	6	6,97	1,90
T2	WW	90	TT/CC	10	10,11	3,43
			CT	6	9,76	4,65
	WS	50	TT/CC	10	7,95	3,68
			CT	6	8,75	4,40
T3	WW	90	TT/CC	10	8,16	3,78
			CT	6	7,36	3,05
	WS	50	TT/CC	10	7,27	3,42
			CT	6	4,44 *	0,98
T4	WW	90	TT/CC	10	7,60	3,86
			CT	6	7,21	2,21
	WS	30	TT/CC	10	2,98	1,32
			CT	6	2,25	0,59
T5	WW	90	TT/CC	10	6,74	3,04
			CT	6	5,87	1,85
	WS	30	TT/CC	10	3,47	1,31
			CT	6	2,57	0,91
T6	WW	90	TT/CC	10	5,34	2,55
			CT	6	4,94	1,31
	WS	30	TT/CC	10	3,55	0,99
			CT	6	3,74	0,81
T7	WW	90	TT/CC	10	5,86	1,89
			CT	6	5,98	0,91
	WS	90	TT/CC	10	4,71	2,21
			CT	6	4,05	1,14
T8	WW	90	TT/CC	10	7,03	2,29
			CT	6	6,76	1,51
	WS	90	TT/CC	10	4,95	1,99
			CT	6	5,76	1,50

Time points	Treatment	FC (%)	SNP chr13_4177522_C_T	Number individuals	Stomatal conductance	
					Mean	SD
T1	WW	90	CT	6	11,70 *	3,28
			CC	9	7,81	2,69
	WS	90	CT	6	10,63	4,03
			CC	9	6,65	1,50
T2	WW	90	CT	6	13,51	4,61
			CC	9	9,46	3,97
	WS	50	CT	6	9,89	3,17
			CC	9	7,83	3,82
T3	WW	90	CT	6	9,32	4,26
			CC	9	7,35	2,61
	WS	50	CT	6	8,69	3,38
			CC	9	4,44 *	0,79
T4	WW	90	CT	6	9,29	3,78
			CC	9	6,71	2,34
	WS	30	CT	6	3,33	1,26
			CC	9	2,46	0,88
T5	WW	90	CT	6	8,05	2,83
			CC	9	5,78	1,81
	WS	30	CT	6	4,01	1,22
			CC	9	2,71 *	0,93
T6	WW	90	CT	6	5,92	2,68
			CC	9	5,08	1,44
	WS	30	CT	6	4,14	0,73
			CC	9	3,47	0,76
T7	WW	90	CT	6	6,75	1,38
			CC	9	5,71	1,17
	WS	90	CT	6	5,62	2,45
			CC	9	3,92	0,95
T8	WW	90	CT	6	8,42	1,77
			CC	9	6,35 *	1,26
	WS	90	CT	6	6,11	1,80
			CC	9	4,91	1,72
