# Supplementary material for: Candidate genes and SNPs associated with stomatal conductance under drought stress in Vitis
Source: BMC Plant Biol. 2021 Jan 6;21:7. doi: 10.1186/s12870-020-02739-z (PMC7789618; doi:10.1186/s12870-020-02739-z)
Supplement: Supplementary file 3 — Additional file 3. [file 12870_2020_2739_MOESM3_ESM.pdf]

Tab. S3 Comparison of *VIT_17s0000g08960* nucleotide diversity in different phenotypic classes.

Parameters	Overall	Rootstocks	Hybrids
Varieties	85	48	37
Number of polymorphic sites	135	109	71
Frequency of polymorphic sites	1:17	1:21	1:33
Synonymous changes	65	49	36
Non-synonymous changes	69	54	34
Synonymous vs non- synonymous mutations	0,9:1	0,9:1	1,1:1
Mean nucleotide diversity (π/θ)	0,007/0,011	0,006/0,011	0,007/0,007
Mean Tajima D	-1,25	-1,36	-0,06
Fu and Li's D	0,29	-1,21	0,27
Shared mutation	52		
Mutation polymorphic only in one group		64	20
