# Supplementary material for: Candidate genes and SNPs associated with stomatal conductance under drought stress in Vitis
Source: BMC Plant Biol. 2021 Jan 6;21:7. doi: 10.1186/s12870-020-02739-z (PMC7789618; doi:10.1186/s12870-020-02739-z)
Supplement: Supplementary file 4 — Additional file 4. [file 12870_2020_2739_MOESM4_ESM.pdf]

Tab. S4 Prediction of tolerability of amino acid exchanges identified in *VIT_17s0000g08960*.

Site	Amino acid change	Provean score	Prediction	Frequency
57	Q19H	-0,473	Neutral	1/85
67	S23A	-0,345	Neutral	1/85
68	S23L	-0,979	Neutral	1/85
74	T25I	-2,617	Deleterious	2/85
91	F31I	-0,114	Neutral	5/85
136	I46F	-3,167	Deleterious	1/85
139	V47M	-1,000	Neutral	16/85
143	A48V	0,521	Neutral	50/85
161	S54I	-0,581	Neutral	64/85
212	A71V	-1,556	Neutral	4/85
217	E73Q	-1,165	Neutral	16/85
227	S76N	-2,181	Neutral	34/85
230	R77L	-4,161	Deleterious	1/85
238	V80I	-0,095	Neutral	1/85
244	V82I	0,696	Neutral	25/85
266	P89R	5,105	Neutral	18/85
266	P89L	-0,338	Neutral	11/85
320	T107S	0,285	Neutral	3/85
340	H114N	-0,508	Neutral	11/85
347	T116N	-4,558	Deleterious	1/85
358	I120L	0,286	Neutral	2/85
380	G127D	-0,352	Neutral	2/85
451	D151N	-1,896	Neutral	1/85
469	V157L	-0,43	Neutral	2/85
478	G160C	-8,205	Deleterious	10/85
494	R165P	-1,325	Neutral	6/85
500	S167F	-2,615	Deleterious	1/85
547	E183K	-0,821	Neutral	6/85
570	K190N	-2,914	Deleterious	6/85
610	E204Q	-1,791	Neutral	4/85
642	F214L	-5,509	Deleterious	1/85
646	W216L	-11,936	Deleterious	1/85
685	E229K	-1,701	Neutral	45/85
687	E229D	-1,211	Neutral	8/85
709	G237S	0,663	Neutral	40/85
736	G246C	-3,552	Deleterious	4/85
796	D266N	-1,031	Neutral	1/85
798	D266E	-0,423	Neutral	5/85
806	G269D	-1,118	Neutral	8/85
863	E288A	-5,362	Deleterious	2/85
904	Q302E	0,394	Neutral	6/85
907	E303K	-0,573	Neutral	4/85
916	M306V	-1,393	Neutral	4/85
946	E316K	-0,299	Neutral	2/85
947	E316V	-3,985	Deleterious	1/85
967	V323L	-2,569	Deleterious	9/85
1016	N339S	-0,021	Neutral	4/85
1031	P344L	-2,591	Deleterious	5/85
1183	S395P	-3,334	Deleterious	1/85
1231	M411L	-0,961	Neutral	1/85
1378	T460S	-0,744	Neutral	1/85
1382	I461T	-1,472	Neutral	26/85
1402	D468N	-4,847	Deleterious	1/85
1643	T548S	0,325	Neutral	13/85
1643	T548I	-2,445	Neutral	1/85
1687	A563S	-1,429	Neutral	2/85
1759	L587I	-1,155	Neutral	4/85
1768	Y590N	-5,534	Neutral	9/85
1768	Y590H	-2,978	Neutral	3/85
1835	K612R	-0,721	Neutral	4/85
1858	T620A	-1,015	Neutral	16/85
1871	L624S	1,004	Neutral	2/85
1878	N626K	-0,634	Neutral	1/85
1964	E655G	-0,536	Neutral	1/85
1986	K662N	-1,504	Neutral	29/85
2039	T680I	-4,523	Deleterious	8/85
2076	N692K	-0,118	Neutral	12/85
2147	E716A	-1,347	Neutral	1/85
2219	E740A	-1,619	Neutral	1/85
