# Supplementary material for: Candidate genes and SNPs associated with stomatal conductance under drought stress in Vitis
Source: BMC Plant Biol. 2021 Jan 6;21:7. doi: 10.1186/s12870-020-02739-z (PMC7789618; doi:10.1186/s12870-020-02739-z)
Supplement: Supplementary file 5 — Additional file 5. [file 12870_2020_2739_MOESM5_ESM.pdf]

Tab. S5 List of primers used for cloning *VIT_17s0000g08960* coding region.

Primer name	Sequence
AMP1 fw	CGCGTTCCCATGTCTTAGC
AMP1 rv	GGTTGTCTCCTACGTGCATG
AMP2 fw	AGCCGGGAGAAGACGACAAC
AMP2 rv	ATTGCAGCCGAGAGTGGAG
AMP3 fw	CTATFFAGGATTTGGCCGTG
AMP3 rv	ACATGGGTGGGTGGATTGAA
AMP4 fw	CCGAACGGCACATTTTGG
AMP4 rv	ACTTGAACCCCAACCGTATG
