# Supplementary material for: Candidate genes and SNPs associated with stomatal conductance under drought stress in Vitis
Source: BMC Plant Biol. 2021 Jan 6;21:7. doi: 10.1186/s12870-020-02739-z (PMC7789618; doi:10.1186/s12870-020-02739-z)
Supplement: Supplementary file 6 — Additional file 6. [file 12870_2020_2739_MOESM6_ESM.pdf]

Tab. S6 List of primers used for quantitative real-time PCR.

Primer name	Sequence
VIT_17s0000g08960 Fw	TGGATATTGGGGTGGGATCAG
VIT_17s0000g08960 Rv	CACGGCCAAATCCTCCATAG
VIT_18s0001g15390 Fw	TGTCCGACTGCCACTTGATC
VIT_18s0001g15390 Rv	GCCCAATGTAGGAGTCCACC
VIT_16s0098g00780 Fw	CACGATGCCCACACCACTAT
VIT_16s0098g00780 Rv	TTTCAAGAGCGCCCAAACCT
VIT_13s0019g03040 Fw	AAGATTGTTGAGCGCCAGAT
VIT_13s0019g03040 Rv	ACGCCATCTGAAAAGGTCAG
VIT_13s0106g00790 Fw	CAAGTACTGGGGCAAGAGGG
VIT_13s0106g00790 Rv	CAGTGGTAGTGGTGCAGAGG
