# Supplementary material for: Candidate genes and SNPs associated with stomatal conductance under drought stress in Vitis
Source: BMC Plant Biol. 2021 Jan 6;21:7. doi: 10.1186/s12870-020-02739-z (PMC7789618; doi:10.1186/s12870-020-02739-z)
Supplement: Supplementary file 7 — Additional file 7. [file 12870_2020_2739_MOESM7_ESM.pdf]

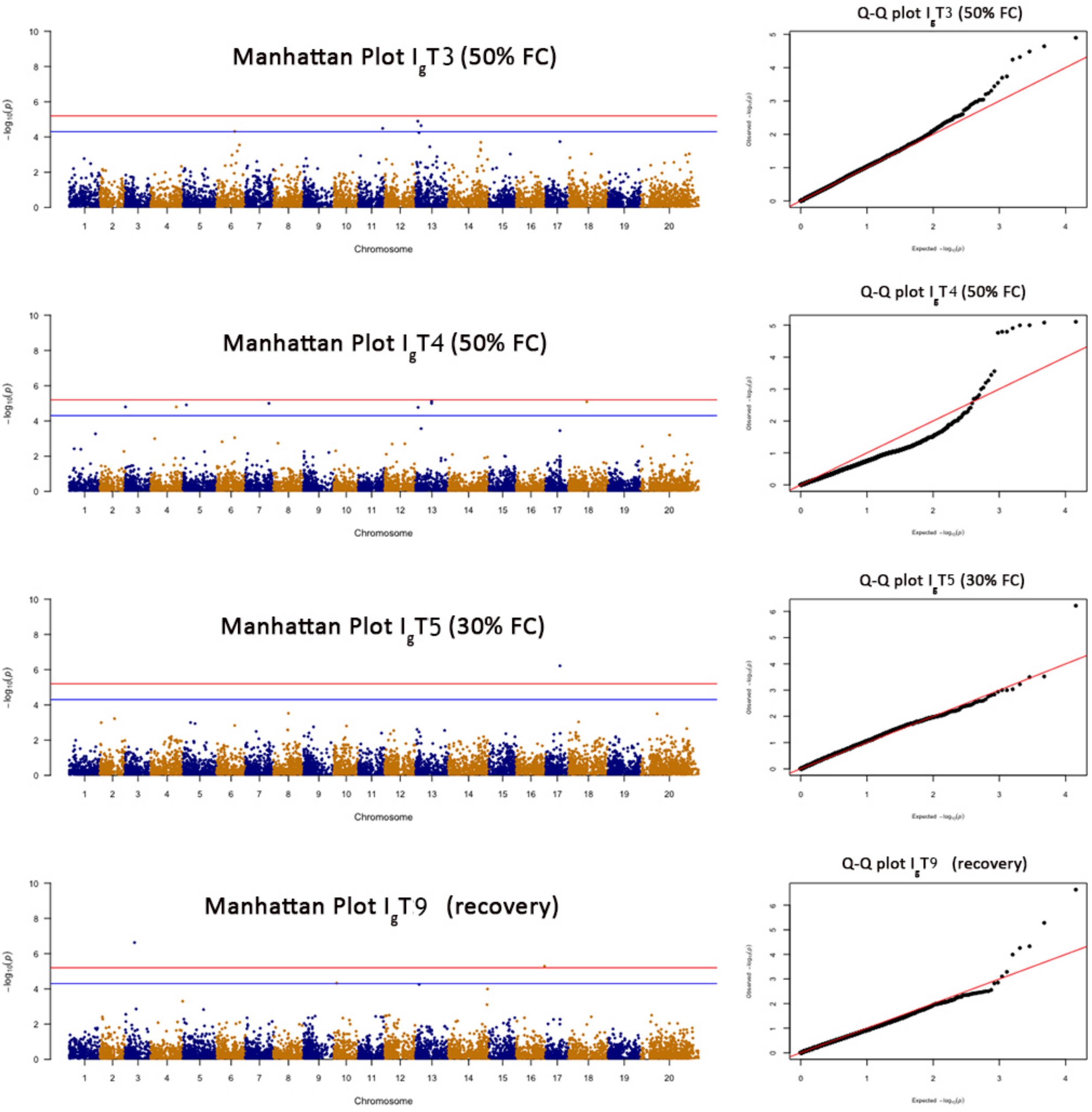

Fig. S1 Manhattan plots and respective quantile–quantile (Q-Q) plots of association analysis between stomatal conductance (Ig) values and all SNP sites at time points T3, T4, T5 and T9 of first year experiment. The log10 P-values are plotted against the position on each of the 20 chromosomes. The horizontal blue and red lines indicate respectively the Bonferroni-corrected p-value and False Discovery Rate (FDR) significance threshold.
