# Supplementary material for: Candidate genes and SNPs associated with stomatal conductance under drought stress in Vitis
Source: BMC Plant Biol. 2021 Jan 6;21:7. doi: 10.1186/s12870-020-02739-z (PMC7789618; doi:10.1186/s12870-020-02739-z)
Supplement: Supplementary file 9 — Additional file 9. [file 12870_2020_2739_MOESM9_ESM.pdf]

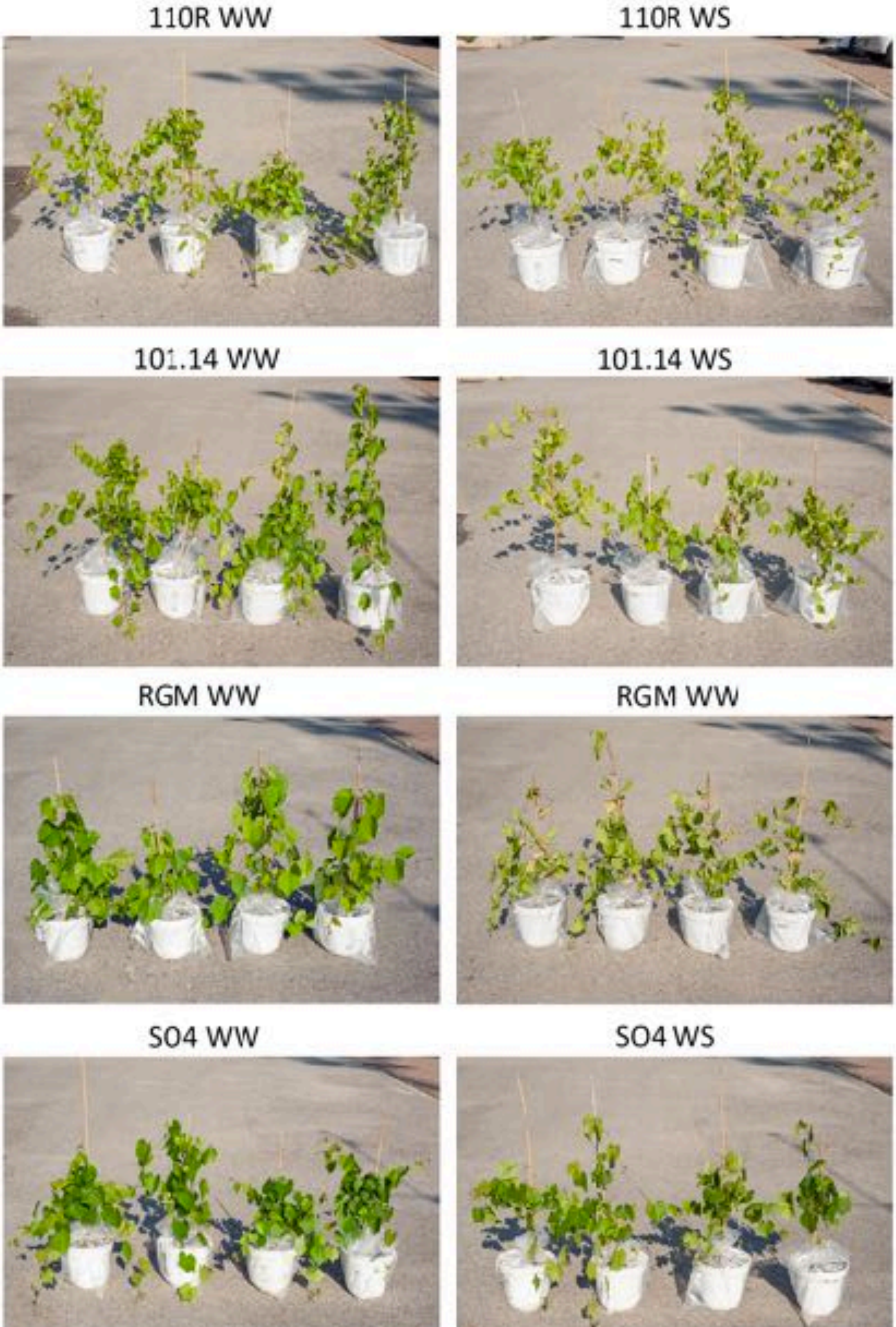

Fig. S3 110R, RGM, 101.14Mgt and SO4 rootstocks of water stress group (WS) and well watered group (WW) at the end of the experiment.
